# A figurational approach to environmental sustainability in the context of sport

**DOI:** 10.3389/fspor.2023.1302458

**Published:** 2023-12-04

**Authors:** Alison Cain

**Affiliations:** Institute of Sport, Psychology, Sport and Geography, Life and Medical Sciences, University of Hertfordshire, Hatfield, United Kingdom

**Keywords:** figurational sociology, environmental sustainability, knowledge transfer, isomorphism, power, policy, unintended consequences

## Abstract

Discourses around environmental sustainability and climate change are increasingly prominent in the sports sector, with a growing range of sports organisations developing policies to address these issues. This paper contends that figurational (or process) sociology can offer a useful framework for examining the development of policy as a process in the context of sport and, specifically, mega-events. The Olympic Games serve as an example for purposes of contextualisation, illustrating four interconnected dimensions of figurational sociology: lengthening chains of interdependence, established-outsider power relations, internalisation of social values, and unintended consequences. Further, the paper seeks to highlight the utility of a figurational perspective particularly when this is enhanced through the integration of complementary concepts, namely knowledge transfer, isomorphism, and diffusion of innovations. Thus, it is asserted that a blended figurational approach can help facilitate understanding of interdependencies and dynamic power relations across expanded stakeholder networks in relation to sports mega-events. Finally, the paper touches on the relevance of sport in relation to the United Nations (UN) Sustainable Development Goals to highlight the need for policy coherence that is arguably unachievable without the understanding of stakeholder interdependencies and power relationships a figurational lens enables. Such understanding is therefore considered to be important as a foundation for the enactment of meaningful policy in the fight against climate change.

## 1. Introduction

Concerns over climate change and a focus on environmental sustainability (ES) are increasingly apparent within multiple and varied sports contexts, with growing recognition that urgent action is needed (1). The expansion of sports stakeholders involved in ES is evidenced by the signatories to the UN Sports for Climate Action Framework (2). This paper offers a perspective on the application of a figurational approach as a means of understanding this expansion and its implications for ES in sport. The focus on ES herein is taken to encompass the climate crisis.

Four conceptual dimensions of figurational (or process) sociology (3, 4) are identified and applied to the topic of ES in the context of sport. These interconnected dimensions are taken to collectively underpin broader dynamic processes of societal development and change (5), which influence the bidirectional relationship between sport and the environment. The discussion focuses on how a figurational lens enables recognition of the complexities that arise in relation to ES policy and sports, as illustrated through the context of the Olympic Games (OG).

The discussion intertwines the selected dimensions of figurational sociology with concepts of knowledge transfer (6), isomorphism (7), and diffusion of innovations (8), which are seen as important in addressing ES and climate issues concerning mega-events and sport policy more broadly. This blended conceptual approach reflects Giulianotti's (9) recommendation that integration of complementary perspectives enhances the application of figurational sociology.

### 1.1. Figurational sociology

Figurational sociology is underpinned by the idea that societies are comprised of social interdependencies whereby people form figurations that extend over time and space, and that dynamic processes and power relations exist within and between these figurations (9). The concept of a figuration exemplifies the relationships and interactions between aspects of society, social institutions (including sport), organisations, and so on. Similarly, the alternative name for this sociological theory, *process*, highlights that societal development and change is processual.

This paper identifies four conceptual dimensions of figurational sociology as being particularly pertinent to ES and sport: lengthening chains of interdependence, established-outsider power relations, internalisation of social values, and unintended consequences (5). The applicability of each of these to sport and ES is contextualised through the OG. In this context, interdependencies exist between an array of people and organisations including the International Olympic Committee (IOC), National Olympic Committees, International Federations, hosts, politicians, athletes, fans, administrators, media companies, equipment and apparel manufacturers, sponsors, environmental pressure groups, and more. As these stakeholder networks grow, the complexity of figurations increases and power relations fluctuate between the various stakeholders.

The four conceptual dimensions are presented in Table 1, with illustrative examples to demonstrate the application of each to the context of ES and the OG. Each dimension is presented separately in Table 1 for purposes of clarity. However, such separation is artificial and unrepresentative of the ways in which the dimensions intersect in dynamic processes of societal development and change, and how these intertwined processes play out in the reality of the sport ES context.

**Table 1:** The application of four dimensions of figurational sociology.

Concept	Application
Lengthening chains of interdependence	This is apparent in the expansion of stakeholders in sport and the hosting of mega-events, for example, growth in size and complexity of the networks (figurations) involved in the Olympic Games in terms of ES initiatives and legacies (6, 10). Figurations include (but are not limited to) the IOC, public authorities, private regulatory bodies, non-governmental organisations, corporate partners, environmental pressure groups, governments (local and national), the public, and the mass media. Multiple stakeholders vary in terms of values, commitment to ES, and power
	Lengthening chains of interdependence allow for enhanced knowledge transfer between stakeholders involved in these figurations
Established-outsider power relations	When established-outsider theory (11) is viewed as a broad theory of power relations it can offer a lens for exploration of the dynamics between the IOC and environmental non-governmental organisations in the development of IOC ES policy and its application to the OG. For example, environmentalist groups becoming insiders in terms of OG preparations as they grow in influence and gain access to decision-making in collaboration with the established IOC (10)
	Since events have, in recent years, been hosted in new geographical regions (12), this extends to the power dynamics between the IOC (established) and host cities (outsiders)
Internalisation of social values	Internalisation of social values leads to regulation of behaviour that may shift to varying degrees between being performative and being authentic. The internalisation of social constraint seen as central to the *civilising process* (4), with accepted modes of behaviour shifting accordingly, may be analogous to evidence of greater authenticity in terms of the IOC commitment to ES as an outcome of the process of showcasing green credentials. In other words, a performative display of care for the environment and the concomitant development of sustainability strategy and policy can gradually result in ES commitment becoming internalised, authentic, second nature
Unintended consequences	There is evidence of unintended consequences resulting from the intentional actions of the IOC, OG hosts and other stakeholders in relation to ES policy development
	Unintended consequences arise from the environmental sustainability policy process—policy development, interpretation, and implementation

## 2. Discussion

As noted above, the interconnectedness of conceptual dimensions is inherent in a figurational approach to understanding the dynamic processes that influence ES in the OG. Thus, the discussion seeks to highlight the interconnected dimensions through select contextualised examples. The interconnectedness means it is not possible (nor appropriate) to discuss each dimension sequentially. The examples presented are not intended as an exhaustive account of the application of figurational sociology to ES and sport, but instead offer a means of highlighting the potential of this perspective. Further, the discussion does not seek to demonstrate all the ways in which a figurational perspective can be combined with related concepts, but is intended to introduce the complementarity of knowledge transfer processes, isomorphism, and diffusion of innovations. This being so, concepts and examples are woven together to provide an overview of the interconnectedness and application rather than to delineate a comprehensive framework.

The impacts of expansion of stakeholders, or lengthening chains of interdependence, involved in mega-events are complex and contested. There are advantages such as the enhancement of isomorphic knowledge transfer opportunities as expertise and experience are shared (13), and disadvantages such as potential for incongruence in terms of policies enacted by different stakeholders (14). Isomorphism is recognised as a process based on legitimacy (8), with organisations seeking to implement policies and practices that have been identified as legitimate in similar settings. Isomorphic processes thus lead to increasing similarity in organisational practices. In the OG context, this is apparent in successive hosts seeking to emulate and exceed the ES commitments of those that preceded them (15). There are three strands of isomorphism, which are all applicable to the context of ES and sport as viewed through a figurational lens (see Table 2).

**Table 2:** Isomorphism and its application.

Form of isomorphism	Examples and integration with figurational dimensions
Coercive Organisations are compelled to adhere to coercive pressures, both formal and informal, exerted by powerful actors in a sector or industry (7)	Coercive isomorphism may be apparent in pressure from NGOs, media, general public (activists), environmental organisations, etc.
	Pressure to adhere to the expectations of stakeholders can be understood through the figurational dimensions of processes of development and change, social values and the consequent regulation of behaviour
	Unintended consequences can arise since actors who are coerced into addressing ES and climate change are not authentically committed to these actions
Mimetic Organisations tend to replicate the policies and practices of others to address uncertainty and, in doing so, homogeneity increases (8)	This includes organisations modelling themselves on others that have been (or appear to have been) successful and are, therefore, deemed legitimate such as Beijing modelling a green bid for the 2008 Games that was based on Sydney's bid for the 2000 Games. From a figurational perspective, this fits with processual development and lengthening chains of interdependence
	Unintended consequences can arise through the cultural misalignment of the modeller and that which is modelled
Normative Organisational practices become subject to regularisation, e.g. through industry standards and certifications, as professional networks expand (7, 8)	This may be seen in the IOC as a global institution of sports governance, or other significant institutions, and the capacity each has to utilise power to achieve ES goals
	This can be understood through a figurational lens in terms of lengthening chains of interdependence (expansion of professional networks) and shifting power relations
	Unintended consequences can arise through differences in the aims and objectives of interdependent social institutions and the power differentials that shape governance organisations and other stakeholders

Mimetic isomorphism is of particular interest here, as it links to knowledge transfer (6) and diffusion principles (8). Moreover, Karamichas (16) refers to the mimetic potential of the OG through internalisation of ES values, which links conceptually with the *civilising process* analogy in Table 1. Additionally, from the figurational standpoint of established-outsider relations, isomorphism represents a process through which outsiders (e.g., a new host of the Games) can seek to integrate with the established (e.g., previous hosts and/or the IOC). Similarly, a nation perceived as being a leader in ES (established) represents a legitimate source of knowledge transfer for a nation seeking to develop in this realm (outsider).

OG host engagement with knowledge transfer processes to learn from and build upon ES initiatives of previous hosts is facilitated to some degree by the IOC (6). Pentifallo and VanWynsberghe (7) identified processes of isomorphism in the motivations of bid committees to exceed the ES objectives of previous OG hosts. Nevertheless, ES progress through transferred knowledge between hosts is not guaranteed. For example, despite knowledge transfer being facilitated through the IOC's Olympic Games Knowledge Management service, the ES outcomes of Rio 2016 were worse than those of previous Games (17–19).

The organisers of Tokyo 2020 sought to build upon knowledge transfer from London 2012 in developing their sustainability plan (20). Tokyo was well-placed to deliver on ES objectives, given the cultural emphasis on *mottainai* and Japan's technological prowess. The Tokyo Games in turn offer opportunities for future knowledge transfer, not only through ES initiatives and related technological innovations, but in terms of response to unforeseen events such as a pandemic. The Covid-19 pandemic necessitated far greater use of remote broadcasting than had been previously implemented for a sports mega-event, which offers opportunities to help reduce climate impacts of future events. Social media can also play a valuable role in disseminating innovation. For example, the use of cardboard beds in the Tokyo athletes' village went viral, and the same initiative is now planned for Paris 2024 (21). Thus, knowledge transfer occurs not only within the sports sector but across domains as innovations are shared, with sports organisations able to draw upon the expertise of diverse stakeholders when planning events. Despite these examples of knowledge transfer, availability of knowledge and innovation does not necessarily mean transfer and implementation will occur.

Successful diffusion of innovations through isomorphic processes is helped or hindered by the importance of ES to the stakeholders involved (8). The IOC's sustainability requirements for host cities should represent an enabling mechanism. However, the potential to enable must be reinforced through monitoring and, if necessary, sanctions for non-compliance, otherwise the constraints imposed by other stakeholders may predominate. Samuel and Stubbs (6) noted that a host city is obliged to fulfil the commitments made and, where this cannot happen, the organising committee can negotiate amendments with the IOC to minimise detrimental impacts. This process of negotiation is framed by the IOC's use of power and leadership in implementing its own ES policy and the ES commitments of hosts. Thus, the IOC should consider how its ES strategy and policy can produce authentic sustainability outcomes rather than simply encouraging hosts to make increasingly bold claims that are not ultimately realised. Since no host has been held accountable for failing to deliver their ES bid promises, the authenticity of IOC declarations of environmental responsibility has been questioned, with inferences of greenwashing (6, 17, 18, 22).

According to Miller (23), the term greenwashing was coined in the mid-1980s to describe hypocritical promises from an organisation that generates ecological harm whilst simultaneously claiming to care about the environment. Sponsorship of sports by organisations that are significant contributors to climate change helps to legitimise those companies and conceal the ecological harm they cause. Miller (23) referred to companies establishing social licenses to operate, which facilitate this legitimisation process. This relates to Corporate Social Responsibility, with companies attempting to appeal to communities in order to minimise or avoid regulation. The process of establishing a social license to operate appears to be hegemonic, with communities becoming complicit in supporting the activities of the organisation in question, regardless of environmental concerns. It is not surprising that sport should be seen as an effective vehicle for the establishment of a social license to operate, a means of legitimisation and greenwashing. In terms of the OG, it has been suggested that the IOC and its sponsors legitimise negative environmental impacts through social licenses to operate (6). These licenses can be established through an emphasis on the benefits for communities of hosting the Games; civic pride, economic benefits, and regeneration. Legitimisation, or at best hegemonic acceptance, of negative environmental impacts is an unintended consequence of the global reach of sport and the expansion of stakeholder networks.

Unintended consequences can result from actions founded on positive intentions. For instance, the short-term measures taken to demonstrate adequate air quality for the Beijing 2008 Games were subsequently considered harmful to genuine and long-term progress on emissions (22). Nevertheless, environmental damage can sometimes lead to positive as well as negative outcomes, both intended and unintended. For example, the environmental degradation associated with the Winter Games of Albertville in 1992 is regarded as having sown the seeds for the incorporation of the environment as the third pillar of Olympism later that decade (24). The potential for positive unintended consequences was noted by McLeod et al. (22), p. 34) in the “awakening of public awareness around environmental aspects of human rights…an opportunity for mass environmental education and…an opportunity for activism”. Certainly, activism on a global scale in relation to the environment has been more prominent in recent years with the activities of groups, such as Extinction Rebellion and Just Stop Oil, drawing attention to the climate crisis and often targeting sports events to gain coverage. Established-outsider tensions are represented by the growing influence of these climate activist groups and the power struggles that are inherent in their actions.

Established-outsider theory (11) has echoes in Howarth's (25) reference to inclusionary and exclusionary processes in the conception of power. He utilises a hegemony approach to understand policymaking, exploring power and discourse in this process. The centrality of power relations in policy development relates to McCullough et al.'s (8) contention that ES initiatives are facilitated or hindered through the importance placed on environmental issues by the stakeholders involved, for example the IOC, OG hosts, NGOs, and others. To extend this, the extent to which ES commitments are performative or authentic is significant in its potential to exacerbate hindrances or enhance facilitation of initiatives aimed at addressing the climate crisis.

The processual development of strategy and policy mean that an organisation's commitment to ES may become increasingly authentic over time and in response to shifting power relations of stakeholders including environmental and political interest groups. That figurational sociology is also known as process sociology offers a cogent argument that it is appropriate for exploring policy development as a process; such appropriateness being akin to what Elias (4) referred to as *object-adequacy*. Thus, it offers a potential explanatory framework for understanding the dynamics involved in any perceived shift towards authenticity regarding ES and the IOC, and how this intersects with changing power relations and lengthening chains of interdependence.

The IOC claims that it “is walking the talk on sustainability” (26), and there are signs of this with a growing range of evidence that the IOC commitment to ES is more than performative, although this is far from undisputable. For example, sustainability principles were embedded into the design, construction, and operation of the IOC headquarters in Lausanne (Olympic House). The building was awarded LEED (Leadership in Energy & Environmental Design) Platinum certification, which confirmed it as one the most sustainable buildings worldwide (27). However, certification is contested territory as it can highlight inconsistencies and result in accusations of organisational hypocrisy (28). From a figurational perspective, it is questionable whether certifications signify internalisation of ES values (being authentic) rather than just virtue-signaling (being performative). Furthermore, the quest for standardised approaches that neglect stakeholder diversity can lead to the unintended consequence of hindering genuine ES progress (28) or may be used as a means of diverting attention away from inaction elsewhere.

Nonetheless, the IOC is becoming more visible in the sport ecology and ES domain, for example through partnering with Sport Positive. The IOC publication of a guide on how to mitigate the impact of sports events on the natural environment (29) and using World Environment Day to reiterate its commitment to sustainability (30) may demonstrate growing authenticity over ES. Reductions in travel also signify internalisation of ES values. For example, ahead of the 2026 Youth Olympic Games, Christophe Dubi (Olympic Games executive director for the IOC) highlighted the importance of taking a hybrid approach and using video conferencing to minimise unnecessary travel by officials (31). This is a positive step when compared to previous criticisms of the IOC's globetrotting.

In view of the leadership capability of the IOC, growing authenticity would have the potential to translate into ensuring ES progress via the application of hegemonic power and isomorphism throughout the figurations of the Olympic Movement. Collective progression in the Olympic family requires shared experiences and dissemination of best practice among bidders, hosts, and other stakeholders, with the IOC leading this as the hub around and through which others interact. The OG could then provide an opportunity (arguably unmatched in scope) for the IOC, host cities, and commercial partners alike to genuinely showcase their green credentials and, perhaps more importantly, reach a global audience with educative and behaviour-changing ideas and ideologies. An ES leadership approach from the IOC that focuses on collaborative engagement among stakeholders would address the complex interdependencies identified by Carswell et al. (32).

With the complex interrelationships that exist in the sports mega-events figurations, collaborative and coherent policy among stakeholders is crucial if the climate crisis is to be addressed and the United Nations (UN) Sustainable Development Goals (SDGs) are to be achieved. The UNs 2030 Agenda for Sustainable Development covers a broad remit of social and environmental issues in its 17 SDGs (33), with at least seven goals that are directly focused on the environment: Clean water and sanitation (no. 6), Affordable and clean energy (no. 7), Sustainable cities and communities (no. 11), Responsible consumption and production (no. 12), Climate action (no. 13), Life below water (no. 14), Life on land (no. 15). Other SDGs, including good health and wellbeing (no. 3) and industry, innovation, and infrastructure (no. 9), are indirectly related to the environment. This broadening of focus to encapsulate more comprehensively the environment and climate reflects growing societal concern with these matters. However, the presentation of these as a set of interlinked goals to be addressed holistically creates a problem in that achievement of one SDG might inhibit achievement of others as unintended consequences occur; as was the case with the Millenium Development Goals (MDGs) that preceded them (34). Furthermore, regarding sport as an enabler of the SDGs, Lindsey and Darby (35) highlighted both the need for policy coherence and the infeasibility of this being achieved across a complex and diverse global sports industry. This reinforces the need for better understanding of interdependencies between stakeholders, power relationships, internalisation of ES values, the potential for unintended consequences, and the development of policy as a process that spans these dimensions. A figurational approach that focuses on these dimensions can facilitate such understanding in the context of sport ES policy and mega-events, especially when combined with complementary concepts such as isomorphic knowledge transfer.

### 2.1. Conclusion

Application of a figurational framework to the study of environmental issues in the sport context can illuminate interdependencies and power relationships among diverse stakeholders and allows for recognition of the potential for ES values to become internalised, shifting from performative to authentic. When combined with related concepts, such as knowledge transfer, isomorphism, and diffusion of innovations, a figurational lens highlights the complexities that exist around sports and ES. Power dynamics and processes of change between established and outsiders in the sport-ES space are illuminated by the integration of these concepts. Although the OG has been used in this paper to illustrate these concepts, the perspective may be similarly applicable to other sports mega-events. As such, a figurational approach may facilitate more nuanced insight into dynamic processes of policy development and enactment as well as policy coherence between various stakeholders in the sports-ES space. Thus, a figurational lens can help develop understanding of the reasons why congruence is present to greater or lesser extents between stakeholder policy and the SDGs. Finally, this approach acknowledges that unintended consequences are inherent in the interactions between stakeholders and arise throughout the policy process, which allows for more authentic and realistic understanding of the potential impact of sport-related ES policies and initiatives in addressing the climate crisis.

## Data Availability

The original contributions presented in the study are included in the article/Supplementary Material, further inquiries can be directed to the corresponding author.
